# Acute Ethanol Exposure Increases the Susceptibility of the Donor Hearts to Ischemia/Reperfusion Injury after Transplantation in Rats

**DOI:** 10.1371/journal.pone.0049237

**Published:** 2012-11-14

**Authors:** Shiliang Li, Sevil Korkmaz, Sivakkanan Loganathan, Alexander Weymann, Tamás Radovits, Enikő Barnucz, Kristóf Hirschberg, Peter Hegedüs, Yan Zhou, Liang Tao, Szabolcs Páli, Gábor Veres, Matthias Karck, Gábor Szabó

**Affiliations:** 1 Department of Cardiac Surgery, University of Heidelberg, Heidelberg, Germany; 2 Heart Center, Semmelweis University, Budapest, Hungary; 3 Department of Cardiac Surgery, Semmelweis University, Budapest, Hungary; 4 Department of Cardiovascular Surgery, Union Hospital, Tongji Medical College, Huazhong University of Science and Technology, Wuhan, China; 5 Department of Otolaryngology, Union Hospital of Tongji Medical College, Hua-Zhong University of Science and Technology, Wuhan, China; 6 Department of Cardiac Surgery, Wuhan Asia Heart Hospital, Wuhan, China; Temple University, United States of America

## Abstract

**Background:**

Many donor organs come from youths involved in alcohol-related accidental death. The use of cardiac allografts for transplantation from donors after acute poisoning is still under discussion while acute ethanol intoxication is associated with myocardial functional and morphological changes. The aims of this work were 1) to evaluate in rats the time-course cardiac effects of acute ethanol-exposure and 2) to explore how its abuse by donors might affect recipients in cardiac pump function after transplantation.

**Methods:**

Rats received saline or ethanol (3.45 g/kg, ip). We evaluated both the mechanical and electrical aspects of cardiac function 1 h, 6 h or 24 h after injection. Plasma cardiac troponin-T and glucose-levels were measured and histological examination of the myocardium was performed. In addition, heart transplantation was performed, in which donors received ethanol 6 h or 24 h prior to explantation. Graft function was measured 1 h or 24 h after transplantation. Myocardial TBARS-concentration was measured; mRNA and protein expression was assessed by quantitative real-time PCR and Western blot, respectively.

**Results:**

Ethanol administration resulted in decreased load-dependent (−34±9%) and load-independent (−33±12%) contractility parameters, LV end-diastolic pressure and elevated blood glucose levels at 1 h, which were reversed to the level of controls after 6 h and 24 h. In contrast to systolic dysfunction, active relaxation and passive stiffness are slowly recovered or sustained during 24 h. Moreover, troponin-T-levels were increased at 1 h, 6 h and 24 h after ethanol injection. ST-segment elevation (+47±10%), elongated QT-interval (+38±4%), enlarged cardiomyocyte, DNA-strand breaks, increased both mRNA and protein levels of superoxide dismutase-1, glutathione peroxydase-4, cytochrome-c-oxidase and metalloproteinase-9 were observed 24 h following ethanol-exposure. After heart transplantation, decreased myocardial contractility and relaxation, oxidative stress and altered protein expression were observed.

**Conclusions:**

These results demonstrate acute alcohol abuse increases the susceptibility of donor hearts to ischemia/reperfusion in a rat heart transplant model even though the global contractile function recovers 6 h after ethanol-administration.

## Introduction

Episodic excessive alcohol consumption commonly referred to as “binge drinking” is common cause of accidental death, violent behaviour as well as suicide, and may be associated with compromised myocardial contractility [1], cardiac arrhythmias, most frequently atrial fibrillation [2] and sudden death [3]. The mechanisms of alcoholic cardiomyopathy, including a) direct cardiotoxicity of ethanol and its major metabolite acetaldehyde, specifically inducing ischemia [4], b) increased production of reactive oxygen species such as hydrogen peroxide [5], c) disturbance in the intracellular calcium homeostasis [6], d) accumulation of fatty acid ethyl esters [7] and e) impaired mitochondrial function may be precipitating events.

Currently, donors with a history of alcohol abuse, accounting for about 1/5^th^ of all donors, are routinely accepted, despite existing evidence supporting the potential deleterious effect of donors' alcohol consumption on recipients' survival and higher rejection rate [8]. Many donor organs come from youths involved in alcohol-related accidental falls, fatal car crashes and suicidal behaviour. Although the success of heart transplantation is highly influenced by good donor selection, the use of cardiac allografts for transplantation from donors after acute poisoning is still under discussion due to potential toxic organ injuries and secondary toxic effects in recipients [9]. Previously some studies have been conducted to evaluate the acute hemodynamic effects of alcohol ingestion in mice, rats and guinea pigs [10], [11]. To our knowledge, in a rat model of potential organ donor, no detailed characterization of the left-ventricular (LV) systolic and diastolic function at various time intervals up to 24 h after alcohol intoxication has been performed *in vivo* using a pressure-volume conductance catheter. Moreover, despite much having been published about the pathogenesis of alcohol intake, molecular mechanisms on the time-course of ethanol-induced cardiac dysfunction are limited. Therefore, the aim of this work was to evaluate the time-course cardiac effects of acute ethanol-exposure and the possible mechanism(s) of action involved in a model of a potential organ donor. In addition, using experimental rat cardiac transplantation, we sought to explore how acute alcohol abuse in donors might affect recipients' cardiac pump function in the early phase after transplantation. This technique includes both reperfusion with blood in an intact animal, simulating the clinical setting, and robust assessment of LV function.

## Materials and Methods

### 1. Animals and Ethics Statement

Male Lewis rats (250 to 350 g; Charles River, Sulzfeld, Germany) were housed in a room at 22**±**2°C under 12-h light/dark cycles and were fed a standard laboratory rat diet and water ad libitum. The rats were acclimatized for at least 1 week before experiments and were randomly assigned to different groups. All animals received humane care in compliance with the *Principles of Laboratory Animal Care* formulated by the National Society for Medical Research and the *Guide for the Care and Use of Laboratory Animals* prepared by the Institute of Laboratory Animal Resources and published by the National Institutes of Health (NIH Publication No. 86-23, revised 1996). This investigation was reviewed and approved by the ethical committee for animal experimentation at Semmelweis University and by the Hungarian government authorities (22.1/2674/3/2011).

### 2. Acute ethanol intoxication

#### Experimental animals

The experimental procedure involved intraperitoneal injections (1 ml/100 g body weight) with either saline (0.9% NaCl) or alcohol. The dose was 3.45 g/kg (75 mmol/kg) body weight for ethanol. Controls were injected with identical volume of saline.

Rats (n = 74) were randomly divided into the following groups: (1) control groups received saline and were euthanized 1 h, 6 h or 24 h after saline injection respectively; (2) ethanol-1 h group (3) ethanol-6 h group and (4) ethanol-24 h group received ethanol and were euthanized 1 h, 6 h or 24 h after ethanol dosing, respectively. We found no difference between the groups receiving saline at different time points with any of the parameters measured and thus combined the values.

#### Electrocardiography

Rats were anesthetized with sodium pentobarbital (60 mg/kg, i.p.) and kept in a supine position on heating pads maintaining their core temperature (measured via a rectal probe) at 37°C. Standard 12-lead electrocardiograms were recorded using needle electrodes placed subcutaneously in each fore leg and hind legs, and six around the chest. All leads were connected to a standard direct-writing recorder (Mortara Instrument, WI, USA). The paper speed was set at 50 mm/sec and the sensitivity at 10 mm/mV. ST-segment elevation and the length of the QT-interval measured in lead-II were the focuses of electrocardiographical analysis. The QT interval was defined as the segment from the onset of the QRS complex to the end of the T wave, defined as the intersection point with the isoelectric line. QT interval was corrected using normalized Bazett's formula adjusted for rats (nQTc = QT/(RR/f)^1/2^) [12]. Electrocardiography was analyzed by an investigator unaware of treatment attribution of the animals.

#### Hemodynamic measurements

Rats were placed on controlled heating pads, core temperature was measured via a rectal probe and was maintained at 37°C, tracheotomized, intubated and artificially ventilated. To assess cardiac function, LV pressure-volume analysis was performed with a 2F pressure-volume conductance catheter (SPR-838, Millar Instruments, Houston, TX, USA). With the special pressure-volume analysis program (PVAN, Millar Instruments, Houston, TX, USA), heart rate, systolic and diastolic blood pressures, mean arterial pressure, LV end-systolic pressure, LV end-diastolic pressure, maximal slope of the systolic pressure increment (dP/dt_max_), maximal slope of the diastolic pressure decrement (dP/dt_min_) as load dependent hemodynamic parameters were calculated. LV pressure-volume relations were measured by transiently occluding the inferior vena cava (reducing preload) under the diaphragm with a cotton-tipped applicator. The slope of dP/dt_max_/end-diastolic volume (dP/dt_max_/EDV) relationship was calculated as load independent index of LV contractility. The slope of the end-diastolic pressure-volume relationship (EDPVR) was calculated as a reliable index of LV stiffness.

At the end of each experiment, 0.1 ml hypertonic saline was injected into the jugular vein, and from the shift of pressure-volume relations parallel conductance volume was calculated by pressure-volume analysis software (PVAN, Millar Instruments, Houston, TX, USA) and used for correction for cardiac mass volume. After completing the hemodynamic measurements, blood samples were collected from the inferior vena cava. The volume calibration of the conductance system was performed as described previously [13]. Nine cylindrical holes in a block 1 cm deep and with known diameter ranging from 2 to 11 mm were filled with fresh heparinized rat blood. The linear volume-conductance regression of the absolute volume in each cylinder in comparison to the raw signal acquired by the conductance catheter was used as the volume calibration formula.

#### Biochemical measurements

Blood collected from the rats in lithium with heparin and gel tubes (S-Monovette®, Sarstedt, Nümbrecht, Germany) was immediately centrifuged and plasma was separated. Levels of plasma cardiac troponin-T and glucose were determined.

#### Histological examination

Hearts from rats in each experimental group were fixed in buffered paraformaldehyde solution (4%) and embedded in paraffin. Then, 5-µm thick sections were placed on adhesive slides and stained with hematoxylin and eosin (H&E). Cardiomyocyte cross-sectional areas were calculated on a microscope (×400) using the Cell∧A software (Olympus Soft Imaging Solutions GmbH, Germany). Histological evaluation was conducted by an investigator unaware of treatment attribution of the animals.

#### Cardiac troponin I immunofluorescence- and Terminal deoxynucleotidyl transferase-mediated dUTP nick end-labeling (TUNEL) stainings

For identification of cardiomyocytes, immunohistochemical staining for cardiac troponin I has been performed. Sections were de-paraffinized with xylene and passaged through decreasing concentrations of ethanol, washed with distilled water and heated with Tris- ethylenediaminetetraacetic acid (EDTA) buffer (pH = 9) for 30 min to retrieve antigenic epitopes. Then, sections were washed with phosphate buffer saline (PBS, 1×) for 3×5 min. After permeabilization by 0.3% Triton X-100 for 10 min and blocking with 1% bovine serum albumin (BSA) and 0.1% Triton X-100, sections were incubated at room temperature for 1 h with the primary rabbit polyclonal antibodies directed against troponin-I (1∶200, Abcam, Cambridge, UK). The incubation with undiluted FITC-conjugated goat anti-rabbit IgG polyclonal secondary antibody (Abcam, Cambridge, UK) was at 37°C for 30 min. After washing with PBS (1×) for 3×5 min, the sections were incubated with 50 µl of Terminal deoxynucleotidyl Transferase (TdT) enzyme and TUNEL Reaction mixture for 1 h at 37°C in the dark (Roche Diagnostics, Mannheim, Germany). The sections were then washed with PBS (1×) for 3×5 min. The slides were mounted using 4′, 6-diamidino-2-phenylindole (DAPI)-Fluoromount-G™ (SouthernBiotech, Birmingham, USA), covered with cover glass and analyzed under a fluorescence microscope. The number of TUNEL-positive cells was expressed as the ratio of DAPI-TUNEL double-labeled nuclei to the total number of nuclei stained with DAPI. Cells were counted in four fields characterizing each specimen), and an average value was calculated for each experimental group. The evaluation was conducted by an investigator blinded to the experimental groups.

#### Quantitative real-time polymerase chain reactions (PCR)

Total RNA was isolated from the hearts with the RNeasy Fibrous Tissue Mini Kit (Qiagen, Hilden, Germany). RNA concentration and purity were determined photometrically (230 nm, 260 nm and 280 nm). Reverse transcription was performed with the QuantiTect Reverse Transcription Kit (Qiagen) using 800 ng RNA in a total volume of 20 µl. Quantitative real-time PCR was performed with the Light-Cycler480 system using the LightCycler480 Probes Master and Universal ProbeLibrary probes (Roche, Mannheim, Germany). The conditions for PCR were as follows: 95°C for 10 min (1-cycle), 95°C for 10 s, 60°C for 30 s (single; 45-cycle quantification), 40°C for 10 s (1-cycle). Efficiency of the PCR-reaction was confirmed with standard curve analysis. Sample quantifications were normalized to glyceraldehyde-3-phosphate dehydrogenase (GAPDH) expression. Primers were obtained from TIB Molbiol (Berlin, Germany), their sequences and UPL probes used are represented on Table 1. Evaluation was performed with the LightCycler480 SW1.5 software (Roche, Mannheim, Germany).

**Table 1:** The sequence for the forward (F) and reverse (R) primers (from 5′ to 3′) and Universal Probe Library (UPL) probes.

Assay	Sequence	UPL probes
Cytochrome c oxidase	F:5′- AGCCAAATCTCCCACTTCC -3′R: 5′- ATAGCTCTCCAAGTGGGATAAGAC -3′	**18**
GAPDH	F:5′- AGCTGGTCATCAATGGGAAA -3′R: 5′- CGGCAGGTCCTTCTCTATCA -3′	**9**
GPx4	F:5′- TGGGAAATGCCATCAAATG -3′R: 5′- CGGCAGGTCCTTCTCTATCA -3′	**25**
Ca_v_1.2, α_1c_-subunit	F:5′- GAGGGCTGGACAGACGTG -3′R:5′- TGACCCTATGATGATTAGTGTTACAAA -3′	**81**
Na^+^-K^+^-ATPase(α_1_-subunit)	F:5′- CACCAAGATAGTGGAGATTCCTTT -3′R:5′- TGGGTTCTTGTGAATGGAGA -3′	**22**
SERCA-2	F:5′- ATGGACGAGACGCTCAAGTT -3′R:5′- GTTTAGGAAGCGGTTACTCCAG -3′	**1**
SOD-1	F:5′- GGTCCAGCGGATGAAGAG -3′R:5′- GGACACATTGGCCACACC - 3′	**5**
MMP-9	F:5′- GGTCAGGTTTAGAGCCACGA -3′R:5′- CCTCTGCATGAAGACGACATAA - 3′	**42**
TNF-α	F:5′- GCCCAGACCCTCACACTC-3′R:5′- CCACTCCAGCTGCTCCTCT-3′	**119**
Inducible NOS	F:5′- CAGCGGCTCCATGACTCT-3′R:5′- ATCTCCTGCATTTCTTCCTGAT-3′	**82**

Glyceraldehyde 3-phosphate dehydrogenase (GAPDH), glutathione peroxidase (GPx)-4, L-Type calcium-channel (α1c-subunit; Ca_v_1.2), sodium-potassium Adenosine Triphosphatase (Na^+^-K^+^-ATPase), sarco(endo)plasmic Ca^2+^-ATPas (SERCA)-2, superoxide dismutase (SOD)-1, matrix metalloproteinase (MMP)-9, tumor necrosis factor (TNF)-α, inducible nitric oxide synthase (NOS).

### 3. Rat model of heart transplantation

#### Experimental groups

Rats (n = 72) were randomly divided into 3 groups for each protocol. First study: (1) 1 h ischemia/1 h reperfusion group (1 h I/R); (2) ethanol-treatment 6 h before explantation followed by 1 h ischemia/1 h reperfusion (6 h ethanol+1 h I/R), and (3) ethanol-treatment 24 h before explantation followed by 1 h ischemia/1 h reperfusion groups (24 h ethanol+1 h I/R).

In the second study, the influence of ethanol exposure on long-term has been investigated: (1) 1 h ischemia/24 h reperfusion group (24 h I/R); (2) ethanol-treatment 6 h before explantation followed by 1 h ischemia/24 h reperfusion (6 h ethanol+24 h I/R), and (3) ethanol-treatment 24 h before explantation followed by 1 h ischemia/24 h reperfusion groups (24 h ethanol+1 h I/R).

#### Method of heart transplantation

Transplantations were performed in isogeneic Lewis to Lewis rat strain, therefore no organ rejection can be expected. The donor-rats of the ethanol groups received a single intraperitoneal injection of ethanol (3.45 g/kg) either 6 h or 24 h prior to explantation. The donor-rats of the control groups received saline instead.

The experimental model was established according to the reported method [14]. Briefly, the donor rats were anaesthetized with intraperitoneal injection of xylazine (3 mg/kg) and ketamine (100 mg/kg) and heparinized (400 IU/kg). A bilateral thoracotomy was performed to expose the heart. After cardiac arrest the superior and inferior caval veins and the pulmonary veins were tied en masse with a 4-0 single silk suture and the heart was stored in cold histidine-tryptophan-ketoglutarate solution (Custodiol, 4°C). The recipient rats were anaesthetized with xylazine and ketamine and then heparinized. The aorta and the pulmonary artery of the donor heart were anastomosed end to side to the abdominal aorta and the vena cava of the recipient rat, respectively. To minimize variability between experiments, the duration of the implantation was standardized at 1 h (ischemic period). After the completion of the anastomoses, the heart was then reperfused with blood in situ for 1 h or 24 h.

#### Hemodynamic measurements

One hour or 24 h after transplantation a 3F latex balloon catheter (Edwards Lifesciences Corporation, Irvine, CA, USA) was introduced into the left ventricle via the apex for administration and withdrawal of fluid to determine dP/dt_max_, dP/dt_min_, developed pressure, LV systolic pressure and LV end-diastolic pressure by a Millar micromanometer (Millar Instruments, Houston, TX, USA) at different LV volumes. From these data LV pressure-volume relationships were constructed using PVAN3.6 software (Millar Instruments, Houston, TX, USA).

#### Thiobarbituric acid reactive substance (TBARS) assay

Heart TBARS concentration was measured by a commercial kit (Zeptometrix Corporation, Buffalo, New York, USA). Briefly, the homogenate was mixed with sodium dodecyl sulfate (SDS) solution and thiobarbituric acid/Buffer Reagent with thorough shaking, and heated for 60 min at 95°C. The samples were then cooled to room temperature in an ice bath for 10 min. The absorbance in the supernatant after centrifugation at 3000 rpm for 15 min was measured at 532 nm using a spectrophotometer (Thermo Electron Corporation, Waltham, Massachusetts, USA).

### 4. Western Blotting

Myocardial proteins were extracted into a solution containing 8 M urea, 5 mM EDTA, 0.002% trasylol, 0.05 mM phenylmethanesulfonylfluoride (PMSF), 0,003% TritonX-100 containing protease inhibitors (Roche, Mannheim, Germany). Protein concentration was determined by a commercial kit according to the manufacturer's protocol (bicinchoninic acid, BCA protein assay kit; Thermo Scientific, Rockford, USA). Total protein homogenates (30 µg) were denatured, separated on sodium dodecyl sulfate polyacrylamide gel electrophoresis (SDS-PAGE) gradient gels (Invitrogen, Darmstadt, Germany) and transferred to polyvinylidene fluoride (PVDF) membrane (Invitrogen, Darmstadt, Germany). The membranes were blocked with 5% milk in Tris-Buffered Saline Tween 20 before incubation overnight at 4°C with primary antibodies specific to superoxide dismutase (SOD)-1 (1∶10000, Abcam, Cambridge, UK), cytochrome-c oxidase (1∶1000, New England Biolabs GmbH, Frankfurt am Main), glutathione peroxidase (GPx)-4 (1∶10000, Abcam, Cambridge, UK), metalloproteinase (MMP)-9 (1∶100, Santa Cruz, Biotehnology, Heidelberg, Germany) and cardiac troponin I (1∶5000, Abcam, Cambridge, UK). After washing blots to remove excessive primary antibody binding, blots were incubated for 1 h with horseradish peroxydase conjugated secondary antibody (1∶5000, Santa Cruz Biothechnology, Heidelberg, Germany). The immunoreactive protein bands were developed using Enhanced Chemiluminescence system (PerkinElmer, Rodgau-Juegesheim, Germany). The intensity of immunoblot bands was detected with a Fujifilm LAS-3000 Imager.

### 5. Chemical reagent

Ethanol absolute, sodium chloride, Triton X-100 were bought from Sigma-Aldrich (Steinheim, Germany). Custodiol was purschased from Dr Franz Köhler Chemie GmbH (Alsbach-Hähnlein, Germany), sodium pentobarbital from Merial GmbH (Halbergmoos, Germany), heparin sodium from Ratiopharm GmbH (Ulm, Germany), buffered paraformaldehyde 4% from Carl Roth GmbH (Karlsruhe, Germany), PBS from Genaxxon Bioscience GmbH (Ulm, Germany), Tris-Buffered Saline Tween 20 from Thermo Fisher Scientific (Cheshire, UK), BSA from Invitrogen Corporation (Auckland, New Zealand), xylene from National Diagnostics (Atlanta, USA), and EDTA from Applied Chem (Darmstadt, Germany).

### 6. Statistical analysis

All data are expressed as means ± standard error of the mean (S.E.M). Means between groups were compared by 1-way ANOVA followed by an unpaired t test with Bonferroni correction for multiple comparisons. A value of p<0.05 was considered statistically significant.

## Results

### 1. Effect of ethanol exposure on the heart

#### Effect of ethanol exposure on cardiac function

**Figure 1:**
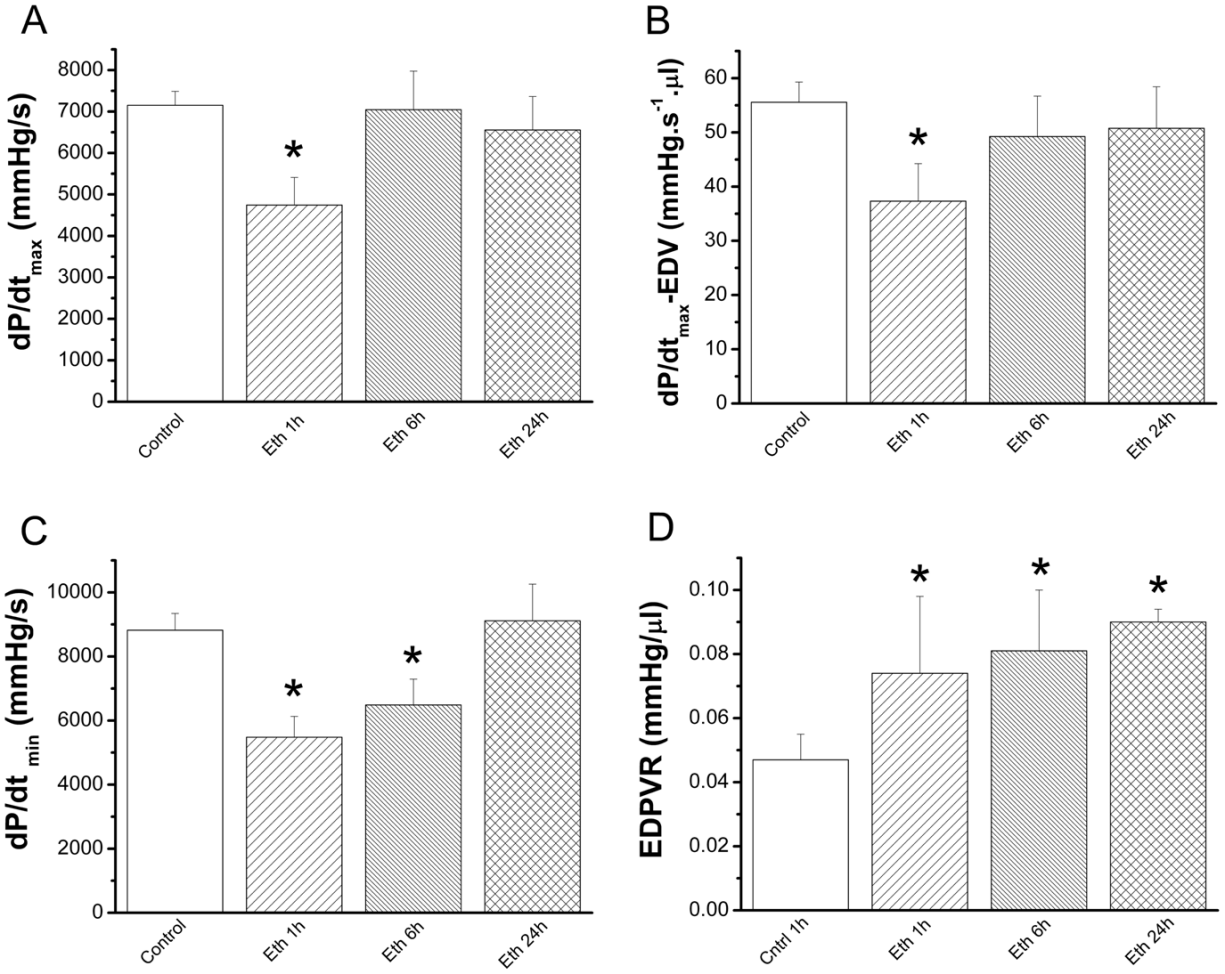
Effect of acute ethanol exposure on cardiac function. Assessment of (A) load-dependent (dP/dt_max_) and (B) load-independent (dP/dt_max_-EDV) contractility parameters revealed a significant decline only at 1 h in response to acute ethanol when compared with the control group. Whereas (C) maximal slope of the diastolic pressure decrement (dP/dt_min_) 1 h and 6 h after ethanol administration was significantly decreased, (D) end-diastolic pressure-volume relationship (EDPVR) was significantly increased in rats treated with ethanol after 1 h, 6 h and 24 h. Eth indicates ethanol. *p<0.05 versus control.

Whereas ethanol exposure had no effect on heart rate, systolic and diastolic blood pressures, mean arterial pressure and LV end-systolic pressure were significantly reduced at 1 h and 6 h and recovered 24 h after ethanol administration compared with the control group (**Table 2**). Assessment of load-dependent (dP/dt_max_), load independent (dP/dt_max_/EDV) contractility parameters, and LV end-diastolic pressure revealed a significant decline only at 1 h in response to acute ethanol when compared with the control group (**Figure 1A–1B** **,** **Table 2**). Whereas 1 h and 6 h after ethanol administration myocardial relaxation (dP/dt_min_) was significantly decreased, EDPVR was significantly increased in rats treated with ethanol after 1 h, 6 h and 24 h, indicating increased end-diastolic stiffness (**Figure 1C–1D**).

**Table 2:** Cardiac hemodynamic parameters.

Parameters	Control	Ethanol 1 h	Ethanol 6 h	Ethanol 24 h
Heart rate, beats/min	345±16	353±23	399±38	390±28
SBP, mmHg	112±4	**80±5** *	**80±7** *	104±7
DBP, mmHg	83±5	**51±4** *	**42±5** *	77±7
MAP, mmHg	93±4	**61±5** *	**59±5** *	86±7
LVESP, mmHg	100±5	**73±7** *	**66±3** *	86±7
LVEDP, mmHg	7.6±0.5	**5.7±0.5** *	6.5±1.1	7.9±1.1

SBP: systolic blood pressure; DBP: diastolic blood pressure; MAP: mean arterial pressure; LVESP: left-ventricular end-systolic pressure; LVEDP: left-ventricular end-diastolic pressure.

* p<0.05 versus control;

# p<0.05 versus ethanol 1 h.

#### Effect of ethanol exposure on ECG parameters and cardiac troponin-T levels

**Figure 2:**
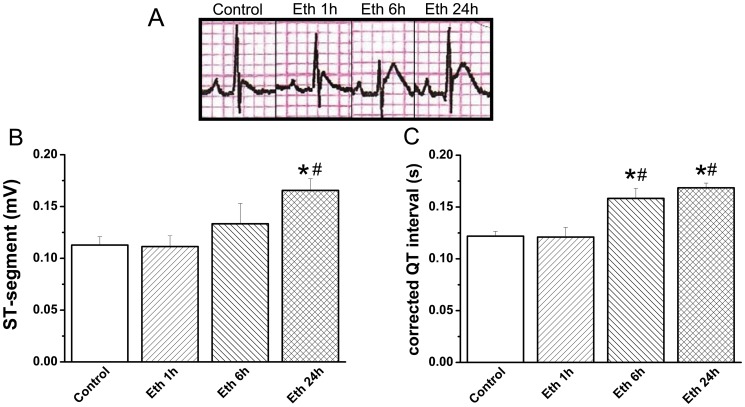
Effect of acute ethanol exposure on electrocardiographic patterns. (A) Representative electrocardiogram tracings. Rats treated with ethanol showed a marked (B) ST-segment elevation at 24 h and (C) elongated corrected QT interval at 6 h and 24 h when compared with the control group. Eth indicates ethanol. *p<0.05 versus control; ^#^p<0.05 versus ethanol 1 h.

Ethanol-treated rats showed a marked elevation in ST-segments at 24 h and elongated corrected QT interval at 6 h and 24 h when compared with the control group (**Figure 2A–2C**). Following ethanol administration, plasma cardiac troponin-T levels remained elevated after 1 h, 6 h and 24 h (**Table 3**).

**Table 3:** Time-course of changes in plasma cardiac troponin-T and glucose levels after acute ethanol administration.

	Control	Ethanol 1 h	Ethanol 6 h	Ethanol 24 h
Cardiac troponin-T, pg/ml	76±11	**201±82** *	**224±73** *	**200±60** *
Glucose, mg/dl	174±5	**343±113** *	227±98	182±20

Following ethanol injection, glucose levels began to increase after 1 h and returned towards baseline values after 6 h and 24 h, cardiac troponin-T levels remained elevated after 1 h, 6 h and 24 h.

* p<0.05 versus control.

#### Effect of ethanol exposure on myocardial histology and DNA strand breaks

**Figure 3:**
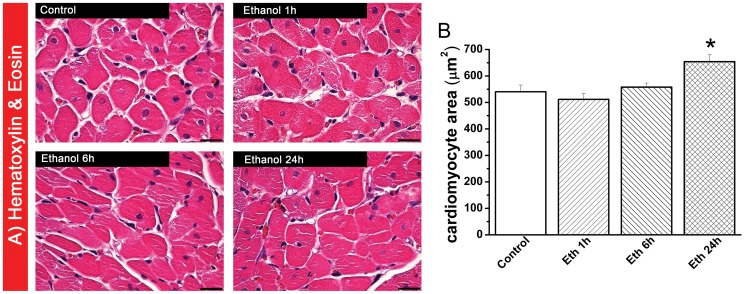
Histological analysis following acute ethanol exposure. (A) Hematoxylin and eosin (H&E) staining micrographs of transverse sections of myocardium (magnification ×400; scale bar: 20 µm) and (B) quantitative analysis of cardiomyocyte cross-sectional area using measurements of ∼20 cardiomyocytes in each group. Twenty-four hours after ethanol administration, the cardiomyocyte transverse cross-section area was significantly increased in the H&E staining sections compared with the control group. Eth indicates ethanol. *p<0.05 versus control.

**Figure 4:**
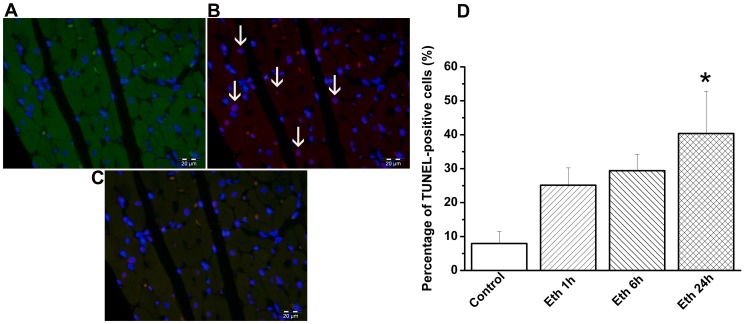
Effect of acute ethanol exposure on DNA-strand breaks in cardiomyocytes. Representative photomicrographs of (A) cardiomyocytes stained with cardiac troponin I (green) and nuclei with 4′, 6-diamidino-2-phenylindole (DAPI, blue), (B) nuclei with fragmented DNA visualized by TUNEL staining (red), and (C) merged image. Magnification ×400; scale bar: 20 µm. White arrows indicate TUNEL-positive cells (not all are marked) (D) Quantification of TUNEL-positive cells. We found DNA damage in the myocardium of rats treated with ethanol at 24 h. Eth indicates ethanol. *p<0.05 versus control.

Histological examination revealed that at 24 h following ethanol administration, cardiomyocyte transverse cross-section area was significantly increased in the H&E-stained sections compared with the control-group and there was no sign of myocardial inflammation (**Figure 3A–3B**). Using TUNEL assay, we found pronounced DNA-damage in the myocardium of rats treated with ethanol at 24 h, as reflected by quantitative assessment of TUNEL-positive cells (containing red fluorescent nuclei), indicating DNA-fragmentation (**Figure 4A–4D**).

#### Effect of ethanol exposure on plasma glucose levels

After ethanol injection, plasma glucose levels began to increase after 1 h and returned towards baseline values after 6 h and 24 h (**Table 3**).

#### Effect of ethanol exposure on relative gene expression

**Figure 5:**
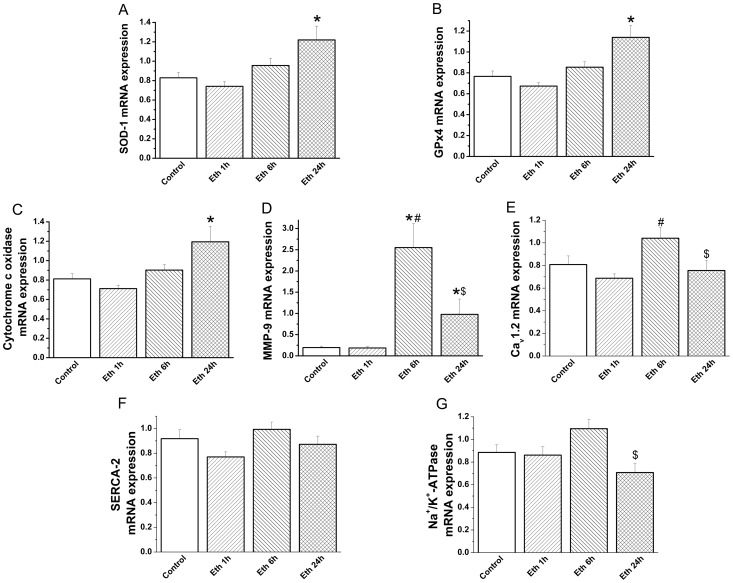
Effect of acute ethanol exposure on myocardial gene expression. Quantitative real-time PCR from myocardium RNA extracts revealed that mRNA levels for (A) superoxide dismutase (SOD)-1, (B) glutathione peroxydase (GPx)-4, (C) cytochrome-c oxidase were significantly increased after 24 h and (D) matrix metalloproteinase (MMP)-9 following 6 h and 24 h compared with the control group. However, mRNA-levels of (E) L-Type calcium-channel (Ca_v_1.2), (F) sarco(endo)plasmic Ca^2+^-ATPase (SERCA)-2 and (G) sodium-potassium Adenosine Triphosphatase (Na^+^/K^+^-ATPase) remained unchanged following ethanol administration compared to the control group. Eth indicates ethanol. *p<0.05 versus control,^#^p<0.05 versus ethanol 1 h, ^$^p<0.05 versus ethanol 6 h.

Administration of ethanol significantly increased SOD-1, GPx-4 and cytochrome-c oxidase relative mRNA expression after 24 h (**Figure 5A–5C**) and MMP-9 mRNA-levels following 6 h and 24 h compared with the control-group (**Figure 5D**). Ethanol administrated rats showed a significant down regulation of TNF-α mRNA expression at 1 h (0.31±0.07 versus 0.71±0.16, p<0.05) and inducible NOS mRNA-levels at 1 h (0.60±0.17 versus 1.14±0.19, p<0.05) and 24 h (0.33±0.06 versus 1.14±0.19, p<0.05) when compared with the control animals. However, mRNA-levels of voltage-gated L-type calcium channel, SERCA-2 and Na^+^/K^+^-ATPase remained unchanged (**Figure 5E–5G**).

#### Effect of ethanol exposure on protein expression

**Figure 6:**
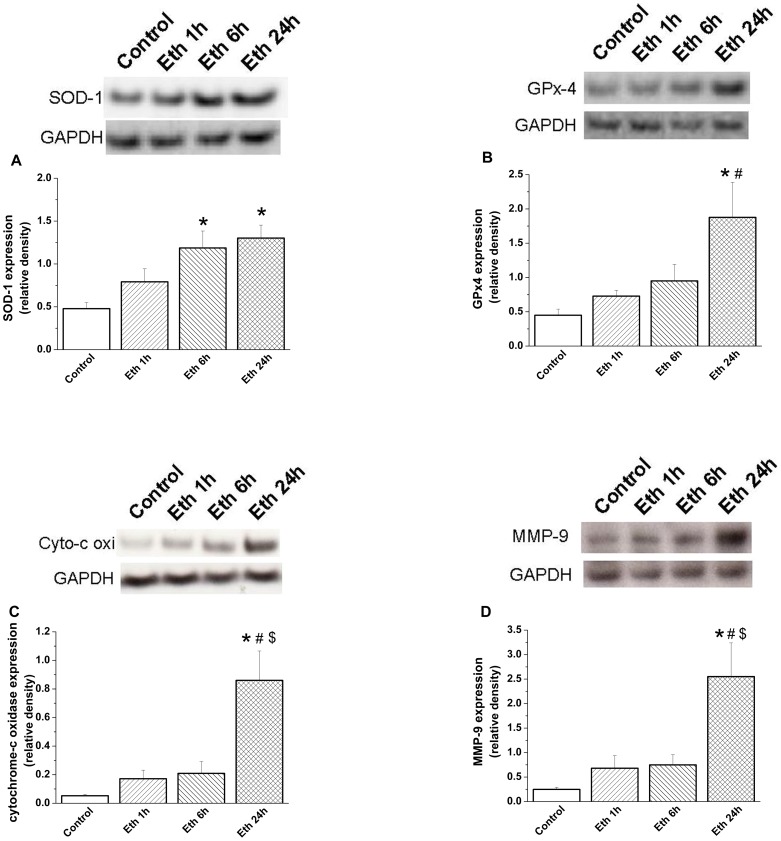
Effect of acute ethanol exposure on myocardial protein expression. Densitometric analysis of bands for (A) superoxide dismutase (SOD)-1 showed a significant increase after 6 h and 24 h ethanol administration, and (B) glutathione peroxidase (GPx)-4, (C) cytochrome-c oxidase (cyto-c oxi) and (D) MMP-9 following 24 h, compared with the control group. Glyceraldehyde-3-phosphate dehydrogenase (GAPDH). Eth indicates ethanol. *p<0.05 versus control; ^#^p<0.05 versus ethanol 1 h, ^$^p<0.05 versus ethanol 6 h.

Densitometric analysis of bands for SOD-1 showed a significant increase at 6 h and 24 h following ethanol administration, and at 24 h for GPx4 (**Figure 6A–6B**), cytochrome-c oxidase and MMP-9 (**Figure 6C–6D**), compared with the control group. However, protein levels of voltage-gated L-type calcium channel, SERCA-2 and Na^+^/K^+^-ATPase remained unchanged (**data not shown**).

### 2. Effect of ethanol exposure on the transplanted heart

#### Effect of ethanol exposure on donor heart function after transplantation

**Figure 7:**
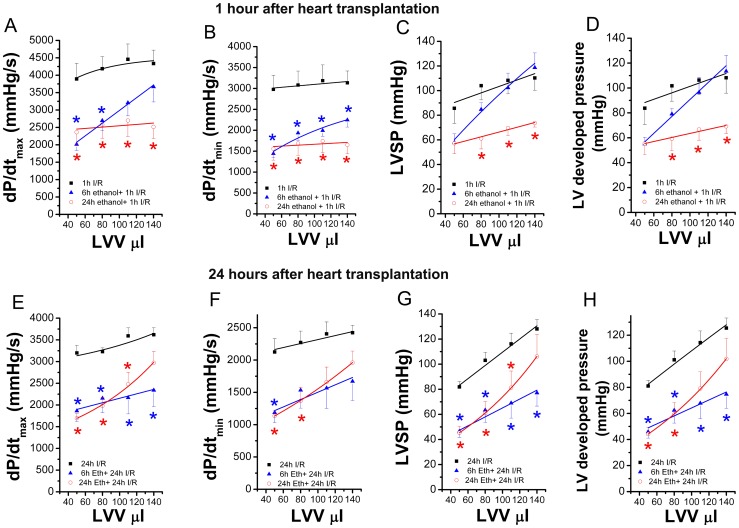
Effect of acute ethanol exposure on donor heart function 1 h or 24 h after transplantation. One hour and 24 h after transplantation, in which donors received ethanol 6 h or 24 h prior to explantation, significantly lower (A, E) dP/dt_max_ (maximal slope of the systolic pressure increment) and (B, F) dP/dt_min_ (maximal slope of the diastolic pressure decrement) were observed when compared with the control-group. Although (C) LV systolic pressure (LVSP) and (D) LV developed pressure, as indexes of myocardial contractile function, were significantly decreased in the 24 h ethanol+1 h I/R group 1 h after transplantation. However, 24 h after transplantation, (G) LVSP and (H) LV developed pressure were significantly decreased in both 6 h ethanol+24 h I/R and 24 h ethanol+24 h I/R groups compared with the control-group. I/R indicates ischemia/reperfusion, LVV left-ventricular volume. *p<0.05 versus 1 h I/R group.

One hour and 24 h after transplantation, in which donors received ethanol 6 h or 24 h prior to explantation, significantly lower dP/dt_max_ and dP/dt_min_ were observed when compared with the control-group, indicating decrease myocardial contractility and relaxation (**Figure 7A–7B** **;** **Figure 7E–7F**). Although LV systolic pressure and LV developed pressure, as indexes of myocardial contractile function, were significantly decreased in the 24 h ethanol+1 h I/R group (**Figure 7C–7D**), LV end-diastolic pressure, as a marker of the standardised balloon-catheter measurements did not show any major differences (**data not shown**) 1 h after transplantation. However, 24 h after transplantation, LV systolic pressure and LV developed pressure were significantly decreased in both 6 h ethanol+24 h I/R and 24 h ethanol+24 h I/R groups compared with the control-group (**Figure 7G–7H**). Ethanol administration had no effect on heart rate 1 h after transplantation (225±11 beats/min 1 h I/R group versus 230±14 beats/min 6 h ethanol+1 h I/R group versus 254±25 beats/min 24 h ethanol+1 h I/R group) and 24 h after transplantation (207±13 beats/min 24 h I/R group versus 230±15 beats/min 6 h ethanol+24 h I/R group versus 211±34 beats/min 24 h ethanol+24 h I/R group).

#### Effect of ethanol exposure on graft TBARS concentration after heart transplantation

**Figure 8:**
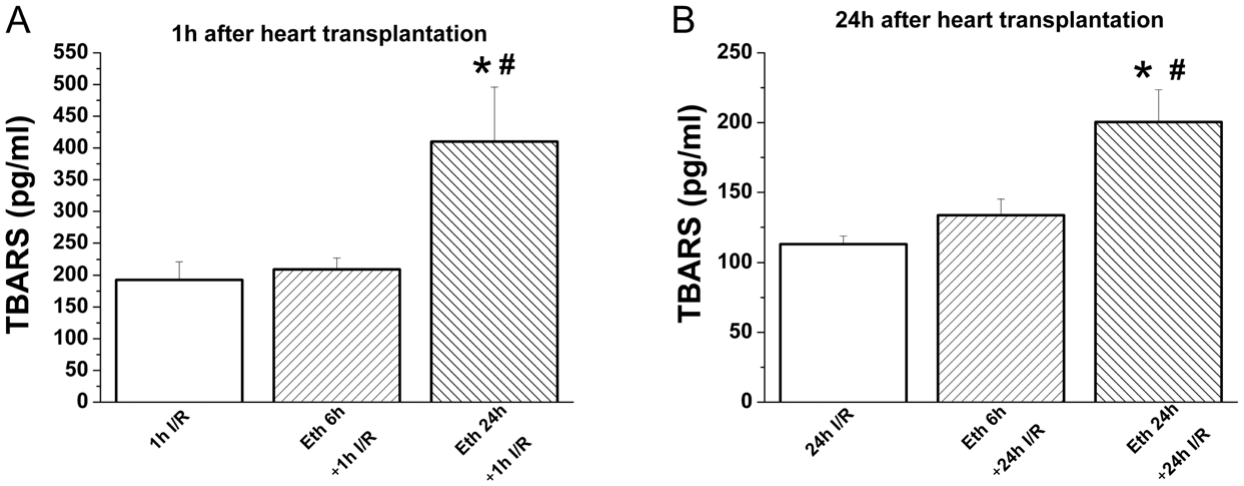
Effect of ethanol exposure on graft thiobarbituric acid reactive substance (TBARS) concentration 1 h and 24 h after heart transplantation. One hour and 24 h after transplantation, there was a significant increase in graft TBARS concentration in the group receiving ethanol 24 h prior to explantation when compared with the control- and 6 h ethanol+24 I/R groups. I/R indicates ischemia/reperfusion, Eth ethanol. *p<0.05 versus I/R groups; ^#^p<0.05 versus 6 h eth + I/R groups.

One hour and 24 h after transplantation, there was a significant increase in graft TBARS concentration in the group receiving ethanol 24 h prior to explantation when compared with the control- and 6 h ethanol+I/R groups (**Figure 8A–8B**).

#### Effect of ethanol exposure on graft protein expression after heart transplantation

**Figure 9:**
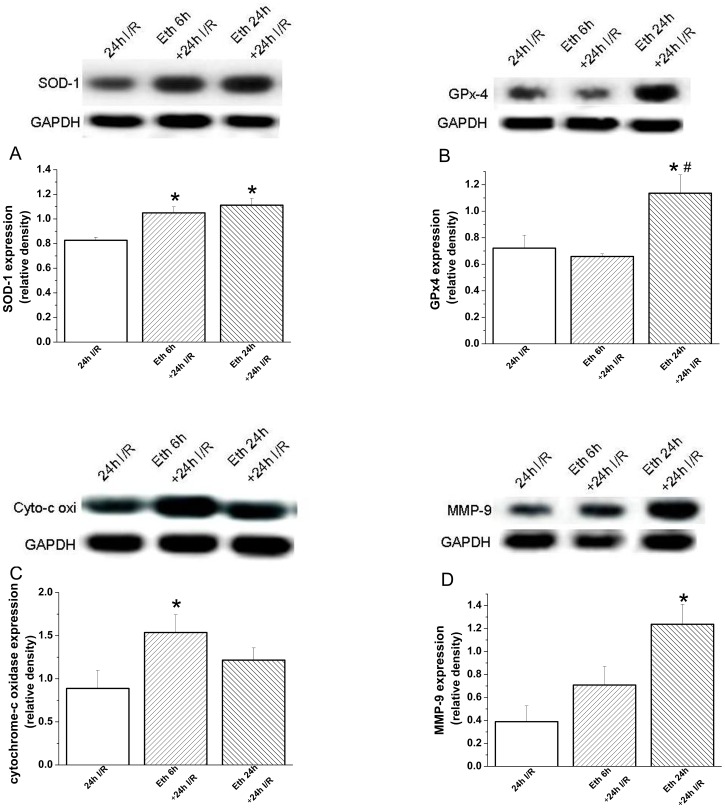
Effect of acute ethanol exposure on myocardial protein expression 24 h after transplantation. Twenty-four hours after transplantation, densitometric analysis of bands for (A) superoxide dismutase (SOD)-1 showed a significant increase at 6 h and 24 h following ethanol administration, at 6 h for (C) cytochrome-c oxidase (cyto-c oxi), and at 24 h for (D) matrix metalloproteinase (MMP)-9 compared with the control group. Moreover, 24 h after transplantation, there was a significant increase in protein expression for (B) glutathione peroxydase (GPx)-4 in the group receiving ethanol 24 h prior to explantation when compared with the control- and 6 h ethanol+24 I/R groups. I/R indicates ischemia/reperfusion, Eth ethanol, glyceraldehyde-3-phosphate dehydrogenase (GAPDH). *p<0.05 versus 24 h I/R group; #p<0.05 versus 6 h Eth+ 24 h I/R group.

Twenty-four hours after transplantation, densitometric analysis of bands for SOD-1 showed a significant increase at 6 h and 24 h following ethanol administration, at 6 h for cytochrome-c oxidase, and at 24 h for MMP-9 compared with the control group (**Figure 9A, 9C, 9D**). Moreover, 24 h after transplantation, there was a significant increase in protein expression for GPx4 in the group receiving ethanol 24 h prior to explantation when compared with the control- and 6 h ethanol+24 I/R groups (**Figure 9B**).

#### Effect of ethanol exposure on graft Troponin I expression after heart transplantation

**Figure 10:**
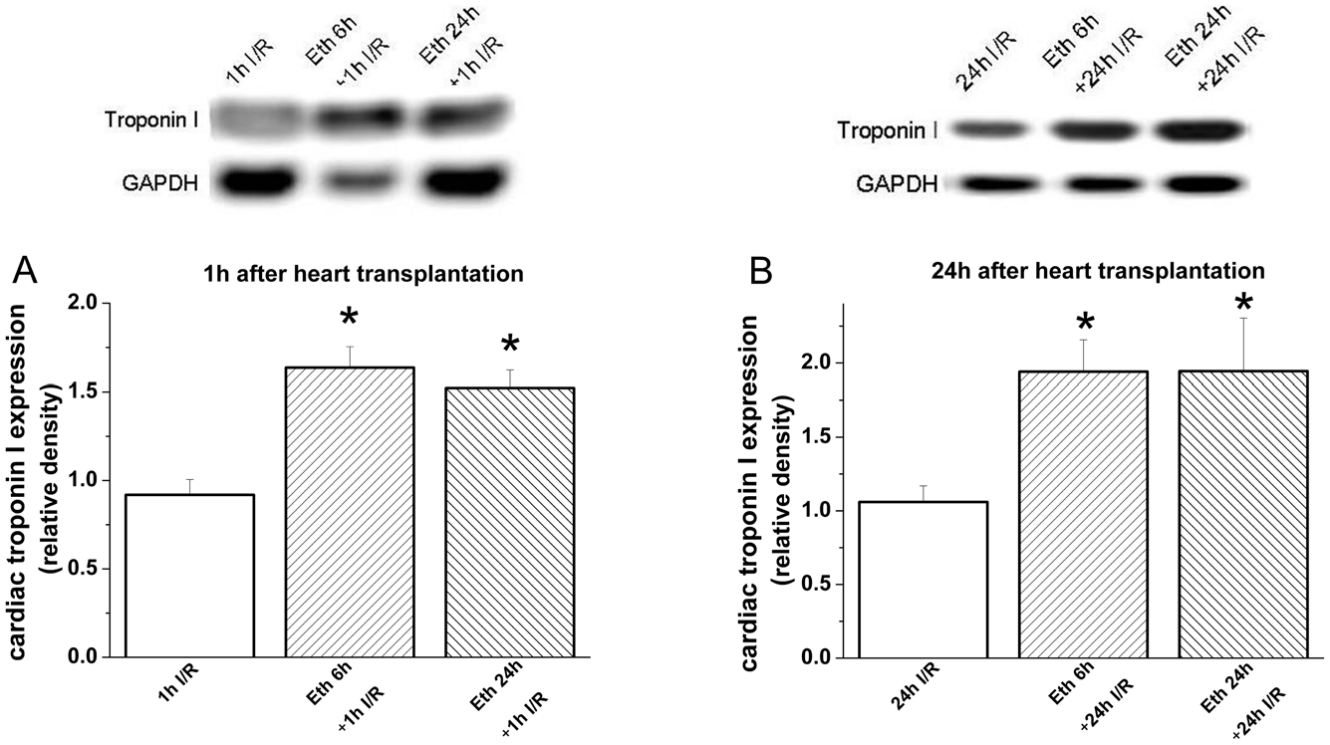
Effect of ethanol exposure on graft Troponin I expression 1 h and 24 h after heart transplantation. (**A**) 1 h and (B) 24 h after transplantation, graft Troponin I expression was significantly increased in the groups receiving ethanol 6 h or 24 h prior to explantation when compared with the control group. I/R indicates ischemia/reperfusion, Eth ethanol *p<0.05 versus I/R groups.

One hour and 24 h after transplantation, graft Troponin I expression was significantly increased in the groups receiving ethanol 6 h or 24 h prior to explantation when compared with the respective control groups (**Figure 10A–10B**).

## Discussion

The major finding of this present study is that acute alcohol abuse increases the susceptibility of donor hearts to ischemia/reperfusion injury in a rat model of heart transplantation even though global heart contractile function recovers 6 h after ethanol administration.

In the present study, we focussed our attention on acute ethanol exposure in which the total consumption is compressed into a short period of time to mimic binge drinking. A bolus of ethanol at a standard dose of 3.45 g/kg body weight, intraperitoneally ensures a complete bioavailability and succeeded in producing rapid rises in circulating level of ethanol. The resulting plasma ethanol concentrations were approximately 375, 185 and 0 mg/100 ml at 1 h, 6 h and 24 h respectively and were similar to pathological levels occurring clinically [15], [16]. It has been shown that ethanol damage to heart becomes evident if alcohol consumption exceeds 90 to 100 g/day in humans [3], which can be transpired to a dosage of ∼1.5 g/kg for an adult weighing 70 kg. In the present study we therefore used the single dose of 3.45 g/kg of ethanol which corresponds to a state of excessive ethanol consumption, given that rodents are less sensitive than human to intoxicating effects of ethanol.

The principal indicator of myopathic alteration following ethanol exposure is characterized by compromised myocardial contractility [3]. This is supported by our results which revealed that after 1 h, ethanol elicited deterioration in the heart function, as evidenced by reduced mean arterial pressure, load-dependent (dP/dt_max_), and load-independent (dP/dt_max_-EDV) contractility indexes. Moreover, we observed decreased LV end-diastolic pressure at 1 h which recovered 6 h and 24 h after ethanol administration. This can be due to the peripheral vasodilatation and relative hypovolemia induced by ethanol, which decreases mean arterial pressure, presumably reduces the LV afterload, and subsequently preload with the consequence that the central venous pressure is lowered. It is conceivable that the systolic function was recovered at 6 h and 24 h when the ethanol levels were either low or absent but not at 1 h. However, the continuation of ethanol's deleterious effects in the absence of circulating plasma levels, suggests that interacting phenomena are occurring. Our data demonstrated that 6 h and 24 h after alcohol administration, myocardial contractility (shown by decreased dP/dt_max_) and relaxation (evidenced by reduced dP/dt_min_) were impaired in the recipients, i.e., these rats show increased susceptibility to myocardial ischemia/reperfusion injury after heart transplantation. Although results of animal experimental studies should not be directly extrapolated to human biology, these novel results have an important implication in cardiac transplantation: acute alcohol intoxication could significantly affect myocardial sensitivity to ischemia/reperfusion even if the donor heart seems to have normal contractile function. In a mouse model of coronary artery occlusion and reperfusion, aldehydes may increase the myocardial susceptibility to ischemia/reperfusion injury [17] and moreover another model of myocardial infarction has shown that with aldehyde dehydrogenase-2 knockout condition, which is accompanied by elevated cardiac acetaldehyde levels, regional ischemia/reperfusion injury was accentuated [18]. The EDPVR reflects the passive properties of the LV and used to obtain a measure of diastolic stiffness. Impaired myocardial relaxation in this study is shown by increased EDPVR for up to 24 h after ethanol, indicating that alcohol administration in rats also induces abnormal diastolic function. Moreover, alterations of active phase of relaxation index (as reflected by decreased dP/dt_min_) were found to recover slowly at 24 h after ethanol administration, when there is no measurable blood alcohol. Taken together, these results suggest that contrary to systolic dysfunction, which was recovered 6 h following ethanol, active relaxation and passive stiffness are slowly improved or sustained during 24 h. We can speculate that the diastolic dysfunction might be at least partially due to the cellular swelling. Due to the direct proportionality of the surface area and myocyte volume, the increase in myocyte profile surface area as evidence in the present work with an enlarged cardiomyocytes size 24 h following ethanol exposure indicates cellular swelling [19].

The donor's cardiac function has been shown to be an important prognostic factoring the clinical outcome of heart transplantation. In the present study we showed elevated circulating plasma cardiac troponin-T levels, the sensitive marker of myocardial injury in heart donors after alcohol intoxication even when hemodynamic measurement showed no evidence of impaired contractile function. A clinical study showed cardiac troponin-T >0.1 µg/l in the serum of heart donors to be predictive of early impaired graft function after transplantation [20]. Previously with the same bolus of ethanol on rat significant elevation in cardiac troponin-T concentration was shown at 2.5 h and 6 h, but at 24 h, in contrast to our results, the levels were not significantly different from controls [21]. It is possible that acute myocardial damage will arise as a consequence of ischemia and occurrence of cardiac arrhythmias. It has been argued that high blood acetaldehyde level might be responsible for the development of myocardial ischemia [4]. In the present study, 24 h after ethanol administration, ischemic damage was assessed histologically by measuring cardiomyocytes swelling and in ECG by ischemic repolarization changes by ST-segment elevation. Alcohol in modest and higher doses has the potential to induce cardiac arrhythmias. The link between alcohol intake and acute disturbances of cardiac electrophysiology has been long suggested. Administration of alcohol in humans even in moderate doses was reported to induce acute prolongation of various electrocardiographic time intervals supporting the evidence of a potential arrhythmogenic effect of ethanol [22]. We observed at 6 h and 24 h following ethanol injection, increased corrected QT-interval, a biomarker for ventricular tachyarrhythmia, which occurred while the heart rate was normal, suggesting that the ventricular repolarization time had lengthened. When the movement of ions across the channels is disturbed, ventricular repolarization is prolonged, resulting in prolongation of the QT-interval. Despite the apparent significance of ethanol interaction with the ion channels on cardiomyocytes membranes, the data published so far describing the effect of ethanol on ionic currents of cardiac cells are scarce. Some authors reported an alteration of calcium homeostasis, especially an up-regulation of cardiac L-type calcium channel in mice exposed to acute alcohol consumption [23]. However, our data showed that following acute ethanol administration both mRNA and protein expression of L-type calcium channel, SERCA-2 and Na^+^/K^+^-ATPase remained unchanged.

Excessive consumption of alcohol over a short period of time (binge drinking) induces a systemic inflammatory reaction [24], which might lead to alcohol-induced myocardial inflammation. Over expression of TNF-α in the myocardium contributes to cardiac dysfunction caused by systemic and local insults. In the present study down-regulation of TNF- α and inducible NOS accounts for the absence of inflammation, which is also supported by our H&E staining showing no sign of myocardial inflammation. It has been shown in monocytes and macrophages that likely acute alcohol exposure directly increases transcription of heat shock protein 70, which in turn could repress TNF-α gene expression [25]. Taken together, decreased LV contractility of the donor heart in the present study seems to be specific to ethanol-induced myocardial injury than inflammation. Moreover, impaired myocardial contractility after transplantation also bolsters this mechanism.

Accumulation of reactive oxygen species in response to ethanol exposure is believed to play an important role. Ethanol or acetaldehyde, the primary metabolic product of ethanol, is known to trigger both oxidative stress and apoptosis [26]. This is also supported by our experimental findings that anti-oxidant enzyme, SOD-1 one of first line defense enzymes mRNA level was upregulated at 24 h and protein level after 6 h and 24 h following acute ethanol exposure. Additionally, both mRNA and protein levels for GPx-4 was increased 24 h after ethanol administration, depicting initiation of oxidative stress. In the present study, we evaluated two antioxidant enzymes, which do not exclude the involvement of other enzymes or non-enzymatic antioxidants following acute ethanol exposure. Moreover, it is known that both *in vivo* and *in vitro*, oxidative stress activates latent resident myocardial matrix MMPs [27]. We showed in this study that MMP-9 mRNA-expression was upregulated at 6 h and 24 h and protein level was increased after 24 h following ethanol administration, which may indicate an increase in oxidative stress. In addition, we showed in this study that acute ethanol exposure resulted in formation of DNA-strand breaks as evidenced by our positive TUNEL staining. Recently, mitochondrial dysfunction also received some attention in the onset of alcoholic complications [28]. Data from our current study revealed that 24 h following acute ethanol administration, there was increased both mRNA and protein expression of myocardial cytochrome-c oxidase, a terminal enzyme in the mitochondrial electron transport chain. This suggests a role of the mitochondrial death pathway in ethanol-induced apoptosis. A previous work showed in an *in vitro* model that isolated cardiomyocytes treated with high-dose of ethanol induced oxidative stress and apoptosis [29]. Consistent also with our data, Guo et al. showed that in mice acute ethanol exposure-induced myocardial dysfunction is associated with mitochondrial damage and apoptosis, supporting an essential role of acetaldehyde and mitochondrial dysfunction in ethanol-elicit alcoholic myopathic alteration [30]. Moreover, ethanol exposure impairs glucose homeostasis. In humans, ethanol consumption is associated with increased circulating glucose concentration, glucose intolerance, and glucose homeostasis [31]. It has been reported that short-term ethanol treatment in rats leads to glucose intolerance similar to that reported in humans [32]. Recent data suggested that chronic alcohol consumption could not only result in glucose intolerance in hepatocytes and skeletal muscles but could also lead to alcoholic cardiomyopathy [33]. In this present study we showed that acute alcohol administration resulted in elevated plasma glucose levels, which peaked at 1 h, time corresponding to decreased cardiac contractility and declined at 6 h and 24 h, times corresponding to normal systolic function of donor hearts.

The use of hearts from donors with a history of “alcohol abuse” remains uncertain [34]. We sought to explore if acute alcohol abuse associated with a potential heart transplant donor can influence recipient graft function because the transplantation of these hearts may unmask abnormalities that may manifest after ischemia/reperfusion injury. The underlying pathophysiological mechanisms of ischemia/reperfusion injury include oxidative stress which could induce apoptosis and mitochondrial dysfunction, changes in calcium homeostatasis, inflammation, osmotic swelling, metabolic modulation, rapid restoration of physiologic pH [35], [36]. In a rat model of heart transplantation, donor rats received ethanol 6 h or 24 h before explantation, these time points were selected to be relevant to clinical cardiac transplantation and the measurements were done 1 h or 24 h after transplantation. Although the explanted heart function was recovered from ethanol-induced systolic dysfunction, we showed impaired contractile function 1 h and also unexpectedly 24 h after heart transplantation. In our laboratory, we previously showed in canine orthotopic heart transplantation [37] and cardiopulmonary bypass models of global ischemia/reperfusion [38] and in a rat model of transplantation-induced ischemia/reperfusion injury [14] decreased LV contractility. However, we and others have already demonstrated that 24 h after transplantation systolic and diastolic function return to normal [39], [40]. In the present study persistence of graft dysfunction 24 h after transplantation needs to be considered during heart transplantation when hearts from donor with alcohol abuse are used. Systolic LV dysfunction is relatively common in even asymptomatic alcoholics, but whether diastolic function is also altered is much less well-studied [41]. Care must be taken because in the present study our data additionally showed that acute alcohol abuse affects diastolic function of donor hearts and the myocardial alterations are at the cellular level. Moreover, in the recipients LV diastolic dysfunction was still present after transplantation. The possible explanation for normal contractile function after 6 h and 24 h following ethanol exposure and depressed systolic and diastolic function after heart transplantation could in part involve the alteration of SOD-1, GPx-4, MMP-9, cytochrome-c oxidase both at mRNA and protein levels due to ethanol exposure. Moreover the increased protein expression levels were still present 24 h after heart transplantation. The changes in calcium homeostasis and the inflammatory process do not seem to play an important role. One hour and also 24 h after heart transplantation, in which donors received ethanol 24 h prior to explantation, an increase in myocardial oxidative stress has been shown as evidenced by elevated myocardial TBARS levels. Thus, all these mentioned events may be further aggravated by ischemia/reperfusion injury with subsequent development of graft dysfunction.

## Conclusions

In summary, in a model of potential organ donor, we demonstrated that after 1 h ethanol induced myocardial contractile dysfunction and elevated plasma glucose concentration. Although these parameters returned to near normal levels 6 h and 24 h after ethanol administration, morphological and molecular changes at the level of myocardium began to appear only at 24 h. Moreover, diastolic dysfunction is also observed following acute ethanol administration and sustained during 24 h. One hour and unexpectedly 24 h after heart transplantation, in which donors received ethanol 6 h or 24 h prior to explantation, decreased myocardial contractility and relaxation were observed even though the global contractile function of the donor hearts recovers 6 h after ethanol-administration. Oxidative stress, apoptosis, and mitochondrial dysfunction could predispose the donor hearts pump function in recipient to increased myocardial susceptibility to ischemia/reperfusion injury after transplantation. Further study is warranted to unveil the impact of acute-on-chronic alcohol ingestion on the outcome of ischemia/reperfusion injury after heart transplantation.

The rat model of heterotopic heart transplantation was selected to be a suitable model to evaluate global ischemia/reperfusion injury. However, this model has certain limitations. In particular, the left ventricle beats in an unloaded condition (e.g. the ventricles are perfused via the coronary circulation, but they do not eject) which on one hand allows a faster recovery after ischemia/reperfusion and on the other hand leads to a time-dependent mechanical deterioration and atrophy. Nevertheless, in detailed characterization studies with this model, it has been shown that major deterioration does not occur until at least 24 h after implantation [39]. Even assessment of long-term effects in the present model was not possible, early graft dysfunction is the main determinant of long-term outcome following transplantation. Therefore, evaluation of graft function on early phase after heart transplantation has clinical significance and importance. Additionally, even though a chronic alcohol ingestion rat model can have more clinically significant scenario, it has been shown that the rat is not very suitable model for studying “alcoholic cardiomyopathy” [42].
